# Study of diesel engine characteristics by adding nanosized zinc oxide and diethyl ether additives in Mahua biodiesel–diesel fuel blend

**DOI:** 10.1038/s41598-020-72150-z

**Published:** 2020-09-18

**Authors:** Manzoore Elahi M. Soudagar, N. R. Banapurmath, Asif Afzal, Nazia Hossain, Muhammad Mujtaba Abbas, Mhd Abd Cader Mhd Haniffa, Bharat Naik, Waqar Ahmed, Sabzoi Nizamuddin, N.M. Mubarak

**Affiliations:** 1 Department of Mechanical Engineering, Faculty of Engineering, University of Malaya, 50603 Kuala Lumpur, Malaysia; 2 Department of Mechanical Engineering, B.V.B. College of Engineering and Technology, KLE Technological University, Vidyanagar, Hubballi, Karnataka, 580031 India; 3 Department of Mechanical Engineering, P. A. College of Engineering (Affiliated to Visvesvaraya Technological University, Belagavi), Mangaluru, 574153 India; 4 School of Engineering, RMIT University, Melbourne, VIC 3000 Australia; 5 Advanced Materials Center, Faculty of Engineering, University of Malaya, 50603 Kuala Lumpur, Malaysia; 6 Department of Mechanical Engineering, Jain College of Engineering, Belagavi, Karnataka, 590014 India; 7 Department of Chemical Engineering, Faculty of Engineering and Science, Curtin University, 98009 Sarawak, Malaysia

**Keywords:** Materials science, Energy science and technology, Fuel cells, Renewable energy

## Abstract

This study deals with an experimental investigation to assess the characteristics of a modified common rail direct injection (CRDI) engine utilizing diesel, Mahua biodiesel, and their blends with synthesized zinc oxide (ZnO) nano additives. The physicochemical properties of diesel, diesel + 30 ppm ZnO nanoparticles (D10030), 20% Mahua biodiesel (MOME20), and Mahua biodiesel (20%) + 30 ppm ZnO nanoparticles (MOME2030) were measured in accordance to the American Society for Testing and Materials standards. The effects of modification of fuel injectors (FI) holes (7-hole FI) and toroidal reentrant combustion chamber (TRCC) piston bowl design on the performance of CRDI using different fuel blends were assessed. For injection timings (IT) and injection opening pressure (IOP) average increase in brake thermal efficiency for fuel blend D10030 and MOME2030 was 9.65% and 16.4%, and 8.83% and 5.06%, respectively. Also, for IT and IOP, the average reductions in brake specific fuel consumption, smoke, carbon monoxide, hydrocarbon and nitrogen oxide emissions for D10030 and MOME2030 were 10.9% and 7.7%, 18.2% and 8.6%, 12.6% and 11.5%, 8.74% and 13.1%, and 5.75% and 7.79%, respectively and 15.5% and 5.06%, 20.33% and 6.20%, 11.12% and 24.8%, 18.32% and 6.29%, and 1.79% and 6.89%, respectively for 7-hole fuel injector and TRCC. The cylinder pressure and heat release rate for D10030 and MOME2030 were enhanced by 6.8% and 17.1%, and 7.35% and 12.28%. The 7-hole fuel injector with the nano fuel blends at an injection timing and pressure of 10° btdc and 900 bar demonstrated the overall improvement of the engine characteristics due to the better air quality for fuel mixing. Similarly, the TRCC cylinder bowl geometry illustrated advanced ignition due to an improved swirl and turbulence. Also, the engine test results demonstrated that 30 ppm of ZnO nanoparticles in Mahua biodiesel (MOME2030) and diesel (D10030) with diethyl ether resulted overall enhancement of CRDI engine characteristics.

## Introduction

Biodiesel is considered as the unsurpassed renewable fuel source due to its’ comparable diesel properties, preparation methods with high yield^1–5^. The other important characteristics of biodiesel include its’ availability, transportability, non-toxic, ease of blending with diesel. Biodiesel provided promising economic viewpoint in the energy market, and positive effect on the environment^6–10^. Despite various advantages, application of biodiesel has several shortcomings, such as low calorific value, poor cold flow properties, and combustion characteristics. Many researchers explored possibilities to enhance the properties of biodiesel; Among them, fuel modification is one the powerful techniques. Fuel modification can be conducted through the addition of nanoscale metallic, carbon, and organic additives^3, 11–15^. The nanoparticles additives in biodiesel improves the oxygen content resulting in complete combustion of the fuel charge, enhancement in heat and mass transfer rates, high catalytic activity, and reduction in the ignition delay (ID) period and harmful emissions^1, 2, 16–21^. Soudagar et al.^1, 2^ studied the effect of aluminum oxide and graphene oxide nanoparticles in honge oil biodiesel (HOME20) and dairy scum oil biodiesel (DSOME20) on a Kirloskar TV1 CMFIS diesel engine. The fuel blends viz. HOME2040 and DSOME2040 showed overall improvement in engine performance and emission characteristics. Also, the combustion characteristics such as peak pressure, cylinder pressure, and heat release rate enhanced while the ignition delay period lowered.

The toroidal re-entrant combustion chamber (TRCC) for fixed compression ratio reduces the HC, smoke, and NOx emission accompanied by increased swirl at TDC due to bulk airflow and turbulence, and improved squish^22, 23^. Enhanced burning in the combustion chamber is achieved by using a piston bowl with large toroidal diameter^24^. The effective management of heat release rate is vital to limit the combustion noise and emission through optimization of the combustion chamber design^22, 25^. Besides, change in the piston bowl diameter to toroidal result in a reduction in BSFC and soot emissions due to the complete combustion of the fuel charge^26^. The toroidal combustion chamber shape also provides better squish, thus, forcing the air to the center of the combustion chamber. The combustion chamber shape results in amplified turbulence, even when the fuel charge is introduced into the combustion chamber^25, 27^. Jyoti et al.^28^ studied the effect of HCC, TCC, and TRCC on a four-stroke single-cylinder diesel engine. The authors observed that due to the swirl motion inside the combustion chamber, there was complete fuel combustion accompanied by the generation of maximum energy. The values of BTE for TCC and TRCC were 2.9% and 3.3% higher than the conventional HCC. The combustion chamber design in a CRDI engine should promote high-intensity air movement in the cylinder with reference to the turbulence, swirl and squish to attain improved air to fuel ratio, higher combustion efficiency and faster evaporation rate.

Recent research on orifice designs of fuel injectors has resulted in the advent of micro orifices. The injector nozzle hole with a small diameter produces droplets of tiny size and results in reduced spray tip penetration due to the low spray momentum. The proper blending of air and fuel droplets is correlated with the diameter and number of nozzle holes^29^. Park et al.^30^ studied the SMD on spray penetration and reported that the fuel injector hole number boosts spray characteristics. The seven injector nozzle holes resulted in complete fuel combustion for different engine models at different speeds^31^.

Deriving motivation from the preliminary investigations on the influence of nanoparticle additives, piston bowl geometries, and fuel injector holes on the diesel engine characteristics has led to its emphasis in the present study. The current research is based on the novel approach by adopting different techniques:

1. The effect of CRDI engine modifications at different injection opening pressures and injection timings are studied:
  - The number of holes in the fuel injectors were varied; 7-hole fuel injector with hole diameter 0.85 mm was used in the present investigation.
  - The toroidal reentrant combustion chamber piston bowl geometry was adopted.
2. The fuel modifications were carried out through the addition of synthesized zinc oxide nanoparticles.
3. Analyses of filter blocking tendency of nano fuel blends were carried out.

Considering the research objectives, an investigation was carried out to determine the physicochemical properties of nano fuel blends. Current research also emphasizes the characterization of synthesized ZnO nanoparticles, ultrasound preparation of nanofuel blends, and transesterification reaction of Mahua oil.

## Results and discussions

### Synthesis and characterization of zinc oxide nanoparticles

The zinc oxide nanoparticles were synthesized using aqueous precipitation method referring to the preceding research by Haniffa et al.^32^. Equations 1 and 2 illustrates the stepwise synthesis of ZnO nanoparticles, 0.5 M of zinc nitrate (Zn(NO_3_)_2_) was added dropwise to 0.5 M of sodium carbonate (Na_2_CO_3_) solution under vigorous stirring.

1 $${\mathrm{Zn}(\mathrm{NO}}_{3}{)}_{2 \left(\mathrm{aq}\right)}+ {{\mathrm{Na}}_{2}{\mathrm{CO}}_{3}}_{\left(\mathrm{aq}\right)}\underset{\mathrm{ RT }}{\to }{\mathrm{ZnCO}}_{3 (\mathrm{s})}\downarrow + {2\mathrm{Na}}_{ (\mathrm{aq})}^{+}+{2\mathrm{NO}}_{3 (\mathrm{aq})}^{-}$$

2 $${\mathrm{ZnCO}}_{3 (\mathrm{s})}\underset{ 650\,^\circ \mathrm{C }}{\to }{\mathrm{ ZnO}}_{(\mathrm{s})}+ {\mathrm{CO}}_{2 (\mathrm{g})}\uparrow.$$

At a temperature of 80 °C and a brief interval of 2 h, the obtained precipitate was then de-humidified in an air circulating oven shortly after the segregation from the mixture. This process was conducted utilizing a vacuum filter with three intervals using condensed water and then ethanol. The dried powder is then retrieved from the oven and was calcined at 500 °C for 3 h to obtain zinc oxide white crystalline nanoparticles. Finally, the nano powder was ball-milled at a speed of 200 rpm for 5 h to obtain fine powder of ZnO nanoparticles.

**Figure 1:**
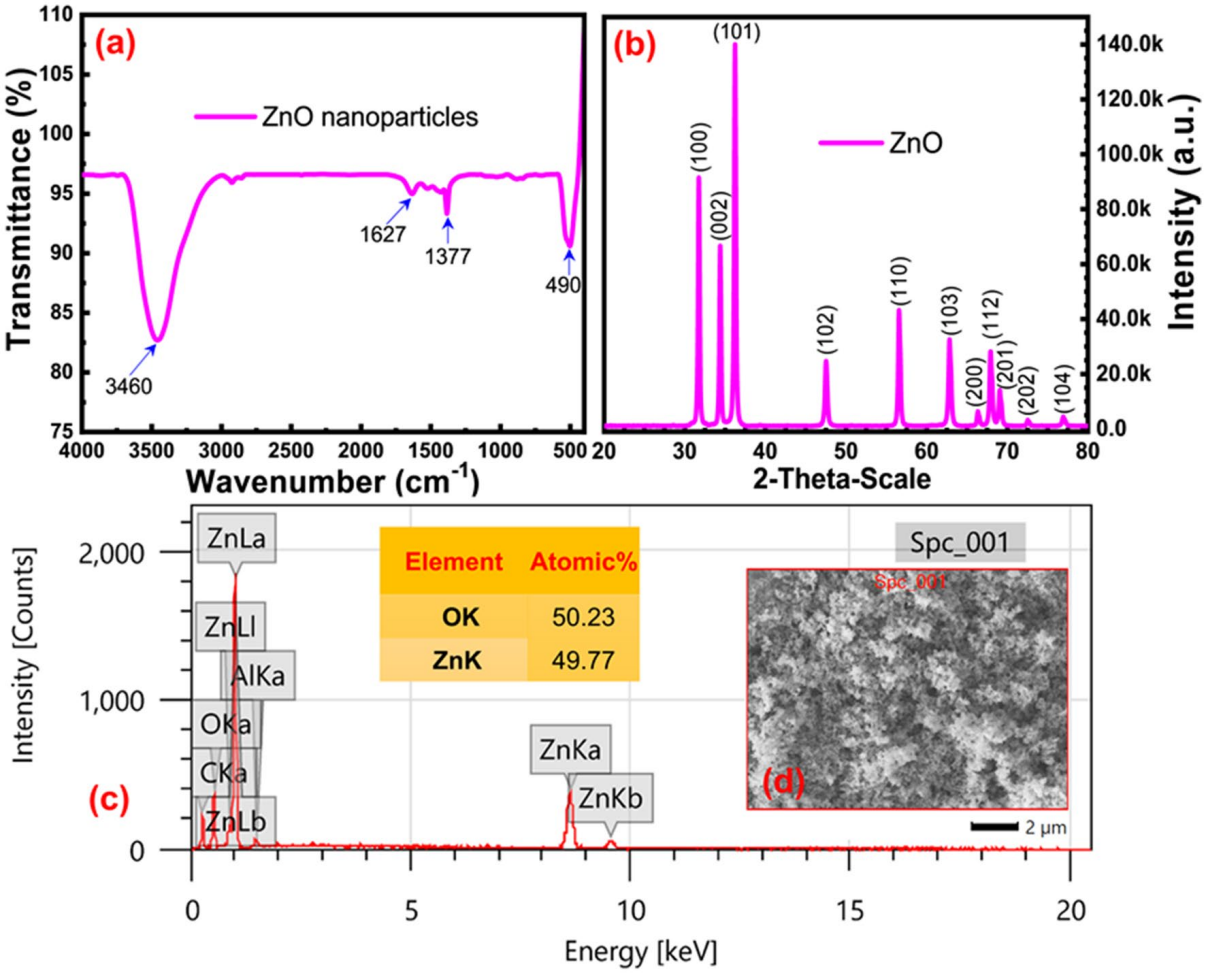
Structural characterization of zinc oxide nanoparticle (**a**) Fourier transform infrared spectroscopy (FTIR) analysis, (**b**) X-ray diffraction (XRD) pattern, (**c**) Energy dispersive X-ray (EDS) analysis.

Figure 1a exhibits the FTIR spectrum of ZnO nanoparticles that indicated two prominent and lower intense peaks along the region from 4,000 to 400 cm^−1^. The corresponding broad peak at 3,460 cm^−1^ was recognized as the stretching vibration of the surface O–H bonds of ZnO nanoparticles. A sharp peak observed at 490 cm^−1^, which can be credited to overlapping the stretching vibrations of Zn–O bonds corresponding to the tetrahedral and octahedral structures of the ZnO nanoparticles. The FTIR from 430 to 420 cm^−1^ assigned to the Zn–O stretching vibration of the tetrahedral structure of the ZnO nanoparticles while the Zn–O stretching vibration of their octahedral structure lies between 540 and 620 cm^−1^. As observed peak assigned to Zn–O stretching vibrations was in good agreement to the previous studies^32, 33^. It was confirmed that in both cases of ZnO nanorods, this extreme Zn–O stretching vibration (490 cm^−1^) rests between 507 and 423 cm^−1^. At the same time, the spherical ZnO NPs illustrated a maximum overlapping at 471 cm^−1^^34, 35^. Furthermore, the FTIR spectrum of ZnO nanoparticle exhibits two lower intense peaks at 1627 and 1,377 cm^−1^ owing to organic contaminations arising from intermediates of a reaction, which is considered as a complex of zinc-hydroxo acetates^36^ or cluster of tetranuclear oxo Zn acetate (Zn_4_O(CH_3_COO)_6_)^37, 38^.

The XRD pattern of synthesized ZnO nanoparticles is illustrated in Fig. 1b, showed distinctive diffraction peaks of ZnO NPs for 2θ values of 31.6, 34.3, 36.8, 48.1, 57.4, 63.2, 66.8, 68.1, 69.3, 73.4, and 77.6 with respect to the corresponding crystallographic planes (100), (002), (101), (102), (110), (103), (200), (112), (201), (202) and (104). Scherrer equation was used to determine the crystallite size recorded to be around 22.5 nm. The XRD pattern of the ZnO nanoparticle exhibited enhancement of the diffraction maxima at 2θ value of 34.3 along with the crystallographic plane (002) direction compared to other directions excluding (100) and (101) (c-axis)^39^. The preferential growth of wurtzite rods was observed through the intensity of the crystallographic plane, and this observation was in agreement with the previous studies^40^. The EDX analysis illustrated in Fig. 1c was carried out using Nova Nano FEG-SEM 450; it was identified that three peaks were representing the existence of the Zn with the sharp and intense peaks at 1.0 keV and weak intense peaks at 0.1 keV, respectively. The oxygen element, a counterpart of Zn atom of ZnO nanoparticle exhibited a peak at 0.5 keV. Besides, a negligible amount of Al and C were observed at corresponding peaks 1.5 keV and 0.8 keV, respectively. These results suggest that the prepared sample contains strong zinc and oxygen signals with a feeble signal of impurities, which may be presented through the precursors. Consequently, it was confirmed the tested sample had high purity of the synthesized ZnO nanoparticles.

**Figure 2:**
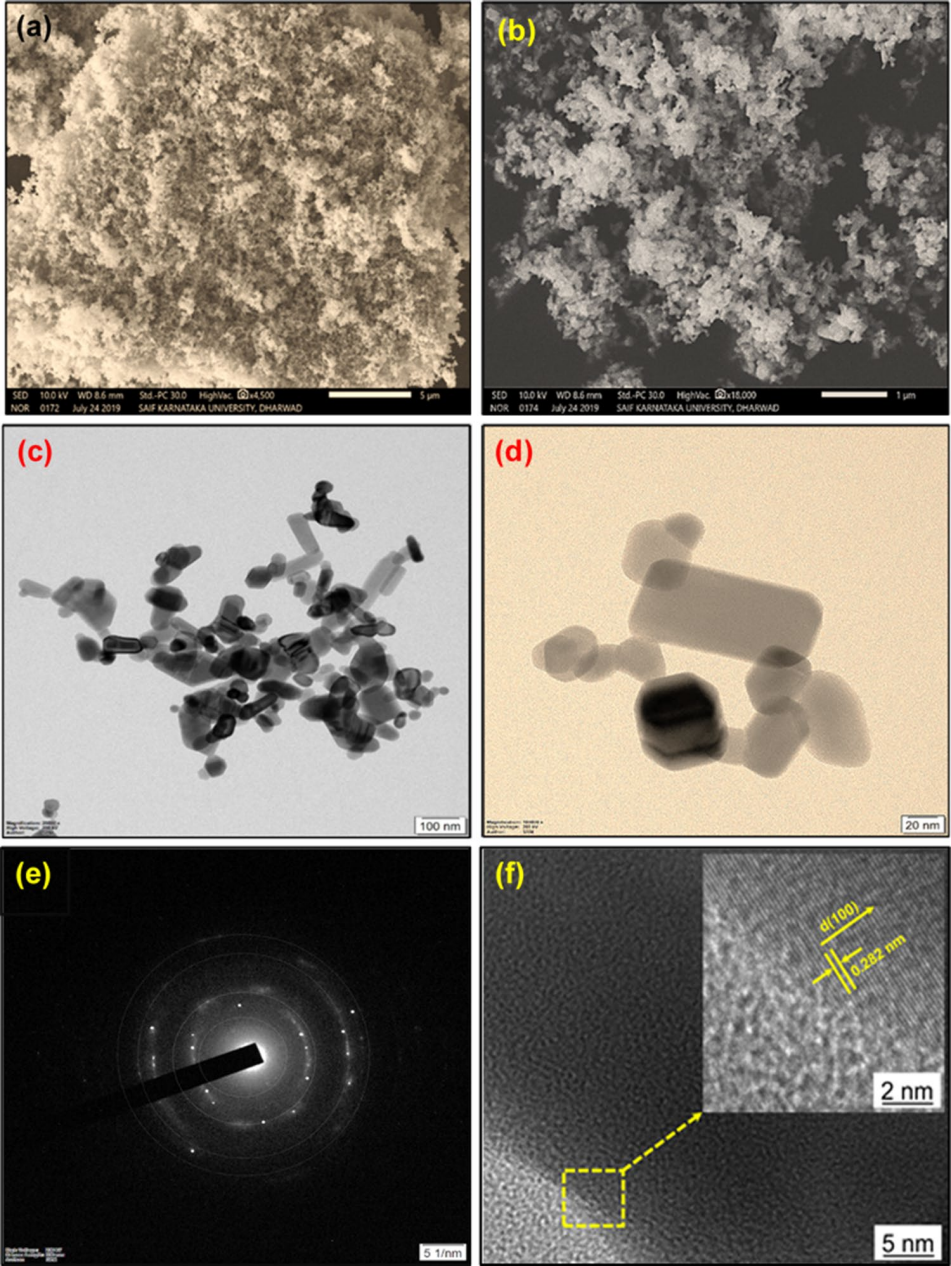
Morphological investigations zinc oxide nanoparticle (**a**,**b**) Scanning electron microscopy (SEM) images, (**c**,**d**) transmission electron microscopy (TEM), (**e**) selective area electron diffraction (SAED) pattern, and (**f**) high resolution transmission electron microscopy (HRTEM).

SEM analysis shows the 3D morphology of ZnO nanoparticles, as illustrated in Fig. 2a,b at 5 and 1 μm magnification levels showing the spherical morphology of ZnO nanoparticles. Figure 2c,d illustrate the TEM images at 100 nm and 20 nm; these confirm the 2-dimensional structures, which include nanorod and spherical shapes and the size of the synthesized ZnO nanoparticles. Besides, interplanar space between the lattice fringes was simulated using HRTEM images as illustrated in Fig. 2f. It was observed that the measured interplanar spacing was 0.282 nm concerning the crystallographic plane (100) and polar c-axis of ZnO nanoparticles. In addition to the XRD pattern, the SAED pattern was used to investigate the crystallinity of the prepared ZnO nanoparticles, as shown in Fig. 2e. The morphological structure of ZnO nanoparticles being smaller than the nozzle hole diameter did not act as a barrier for the fuel flow.

### Analysis of uncertainty

The uncertainty analysis comprises of the mean of repeat measurements to estimate the actual value. The average of three readings of a selected parameter were considered for the error analysis^1, 2^. The error bars were represented for all engine characteristics to indicate the uncertainty in measurement.

The percentage of uncertainties of the calculated and measured parameters are demonstrated in Table 1.

**Table 1:** Accuracies and the uncertainties in the calculated parameters.

Device specifications/parameters	Range	Accuracy	Percentage uncertainties
Smoke meter AVL 4,000	0–100 HSU	± 0.1 HSU	± 0.5
Fuel flow meter	1–30 cc	± 0.1 cc	± 0.9
Crank angle encoder	0–720°CA	± 0.2°CA BTDC	± 0.4
Speed tachometer	0–10,000 rpm	± 5 rpm	± 0.1
Torque	0–80 N m	± 0.05 N m	± 0.08
Pressure transducer (bar)	0–100 bar	± 0.1 bar	± 0.2
Brake thermal efficiency	–	–	± 0.8
Brake specific fuel consumption	–	–	± 0.9
Eddy current dynamometer	0.2–8 kW	0.015 kW	± 0.6
Kinematic viscosity	0.2–20,000 mm^2^/s	–	± 0.5
Density	0.65–3.0 g/cm^3^	0.0001 g/cm^3^	± 0.03
CO emission	0–10 vol.%	± 0.01 vol.%	± 0.07
NOx emission	0–4,800 ppm	± 10 ppm	± 0.7
HC emission	0–30,000 ppm	± 15 ppm vol	± 0.8
Power	0–92 kW	± 0.07 kW	± 0.1
Digital stopwatch	–	± 0.1 s	± 0.2
Heat release rate	–	–	± 0.5
Exhaust gas temperature	0–900 °C	± 1 °C	± 0.4

### Properties of fuel blends

The research conditions, environment, and equipment were followed by the preliminary investigations by Soudagar et al.^1, 2, 41^. Table 2 demonstrates the properties of the diesel (D100), biodiesel (MOME20), and nanofuel blends (D10030 and MOME2030). The free fatty acid content in fuel influences the kinematic viscosity of a fuel blend. The kinematic viscosity of MOME20 was higher than other fuel blends; the nanofuel blends illustrated a slight reduction in viscosity as a result of the addition of 2 vol.% DEE. The diesel fuel demonstrated the lowest viscosity due to the absence of ZnO nanoparticles. The calorific value of D10030 and MOME2030 fuel blends increases due to the addition of ZnO nanoparticles. Also, the nanofuel blends illustrated enhanced cold flow properties.

**Table 2:** Properties of the diesel, biodiesel, and nanofuel blends.

Properties	Unit	ASTM standards	Test limit ASTM D6751	Diesel^a^	MOME20^a^	D30^a^	MOME2030^a^
Density	kg/m^3^ at 15 °C	D4052	860–900	836.6	850	840.5	845.2
Calorific value	kJ/kg	D5865	Min. 35,000	42,800	41,950	43,200	42,800
Kinematic viscosity	cSt at 40 °C	D445	1.9–6	2.4	3.82	2.95	3.78
Specific gravity	gm/cc	D891	0.87–0.90	0.82	0.94	0.85	0.90
Cetane number	–	D613	Min. 40	48	52.5	49	53.6
Flash point	°C	D93	> 130	80.9	95	78.65	90.5
Pour point	°C	D97-12	− 15 to 16	− 7	4	− 1.5	3.5
Cloud point	°C	D2500-11	− 3 to 12	− 2	6.5	1	5.8
Sulphur content	% w/w	D5453	0.05	0.007	0.100	0.010	0.016
Water content	vol.%	D2709	0.05 vol.%	–	Trace	–	Trace
Carbon residue	wt%^5^	D4530	0.050 wt%^5^	0.040	0.160	0.080	0.100
Copper strip corrosion	–	D130	Max. 3	1	–	–	–

^a^Analyzed results.

### The effect of different factors influencing the engine combustion characteristics

**Figure 3:**
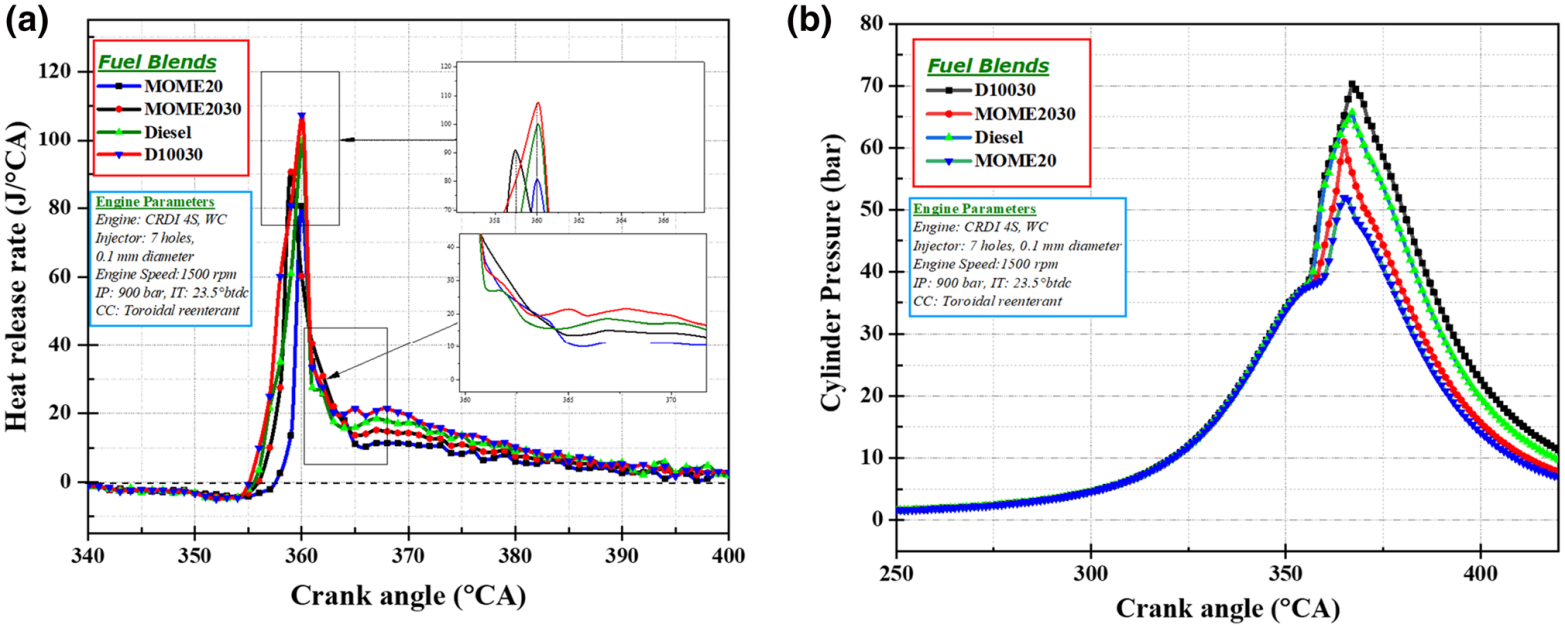
Variation of (**a**) heat release rate and (**b**) cylinder pressure at different crank angles.

This section deals with the impact on piston bowl geometry, fuel injector holes, and nanofuel blends on engine combustion characteristics. The heat release rate (HRR) and cylinder pressure were analyzed for the 7-hole injector at maximum loads. These parameters illustrate the effect of a higher number of holes and TRCC on the combustion characteristics of a CRDI engine fueled with diesel, biodiesel and nanofuel blends. The ZnO nanoparticles release more heat of the combustion for test fuel due to the high thermal conductivity and better thermal stability. The heat release rate was determined using Heywood’s mathematical equation. Equation 3 illustrates the heat release rate model adopted in the current study,

3 $$\frac{{dQ_{total} }}{d\theta } = \left( {\frac{{\gamma_{s} }}{{\gamma_{s} - 1}}} \right)\left( {P_{c} } \right)\left( {\frac{dV}{{d\theta }}} \right) + \left( {\frac{1}{{\gamma_{s} - 1}}} \right)\left( V \right)\left( {\frac{dP}{{d\theta }}} \right) + \left( {\frac{{dQ_{w} }}{d\theta }} \right)$$

where $\frac{d{Q}_{total}}{d\theta }$ indicates the heat release rate, P_c_ and *γ*_*s*_ shows the cylinder pressure and specific heat ratio, $\left(\frac{d{Q}_{w}}{d\theta }\right)$ and V illustrates the heat transfer rate from the gases to the cylinder wall and volume of the combustion chamber. Figure 3a shows the variation of HRR at different crank angles.

When the nano fuels are injected in the combustion chamber, it receives excess heat from the thermally active ZnO nanoparticles resulting in the early ignition of the test fuel. The 7-hole fuel injector and TRCC demonstrated a higher heat release rate for all fuel blends due to the better air–fuel mixing and development of effective swirl motion. The HRR for MOME20 fuel blend turned out to be the lowest in contrast to the diesel fuel on account of higher molecular mass and lower laminar burning velocity. When the combustion starts, the HRR turns positive, and consequently, rapid burning of fuel blends occurs during the premixed combustion phase resulting in a higher heat release rate. The heat release rates for the diesel and D10030 fuel were 99.5 J°/CA and 107.5 J°/CA, respectively. The nano-diesel fuel illustrated enhanced HRR compared to other fuels blends because of the combined physicochemical properties of diesel DEE and ZnO nanoparticles. This approach leads to an improved hear transfer rate, high thermal conductivity, and lower viscosity. The zinc oxide nanoparticles in MOME2030 fuel blend lead to an increase in the fuel cetane number and reduced ignition delay period, the HRR for MOME2030 (90.7 J°/CA) was comparable to D100. The fuel blend, MOME20 illustrated lower HRR (80.6 J°/CA) compared to all other fuel blends due to the poor spray atomization, weak volatility, higher viscosity and surface tension, and density. Figure 3b illustrates the cylinder pressure for test fuel blends at maximum load for a 7-hole fuel injector. At all, crank angles for TRCC and 7-hole FI due to better air and fuel mixing and high activation energy of ZnO nanoparticles that result in an enhanced swirl and squish movement within the piston bowl^42^. The viscosity and lower heating magnitude of the MOME20 lower the cylinder pressure. Hence, a maximum cylinder pressure of 51.9 bar was observed for MOME20 at 365°CA. At maximum load, the cylinder pressure found for MOME2030 (MOME20 + 30 ppm ZnO) was 57.9 bar, the cylinder pressure improves due to the catalytic effect, shorter ignition delay, the higher surface area of ZnO nanoparticles^43–45^.

### The effect of injector opening pressure (IOP) on engine characteristics

#### Effect of injector opening pressure (IOP) on engine performance characteristics

**Figure 4:**
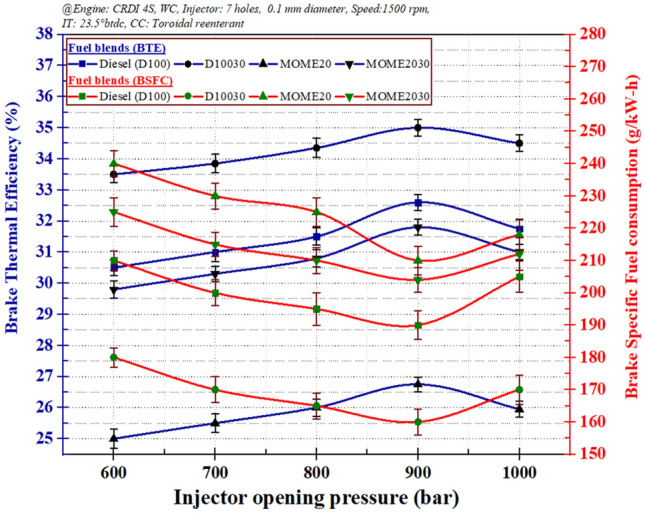
The variation of injection opening pressure: (**a**) brake specific fuel consumption, and (**b**) brake thermal efficiency.

Figure 4 shows BSFC and BTE for a 7-hole fuel injector at 80% load at different IOP. The BSFC for diesel and other fuels followed a common trend, wherein the fuel consumption steadily reduces with an increase in pressure from 600 to 900 bar.

The increasing IOP provides excellent combustion of the fuel up to a specific upper limit. After 900 bar, any further increase in injection pressure reduced BTE and increased BSFC. The reason could be the nature of fuel spray momentum into the compressed air density, this leads to the requirement for more fuel for the same power output. Hence, an increase in injection pressure causes more efficient fuel combustion up to a specific limit beyond the condition while the injection of fuel that increases performance^45–47^. The high viscosity and lower calorific value of MOME20 was another reason for lower BTE^48, 49^.

##### Statistical analysis for performance parameter and injection opening pressure

**Figure 5:**
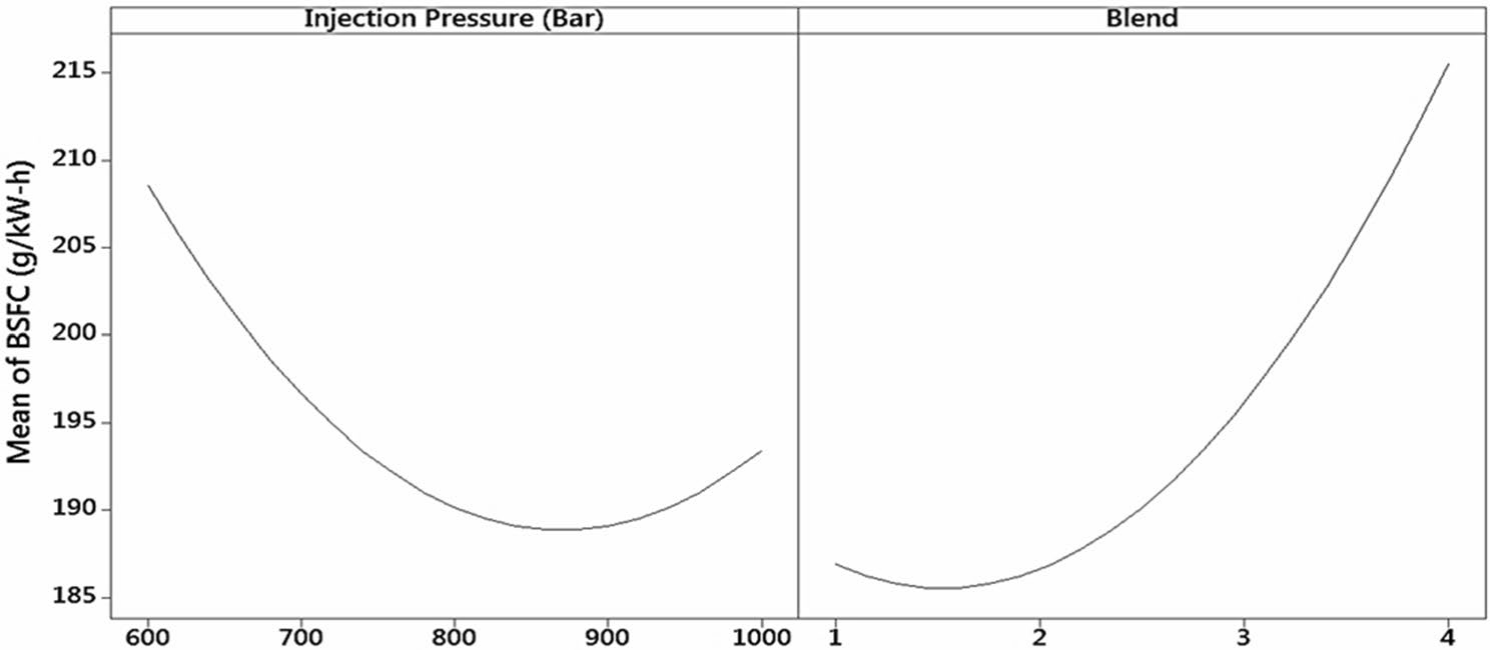
Main effects on plot of mean brake specific fuel consumption (BSFC) by injection opening pressure and blend on BSFC.

**Figure 6:**
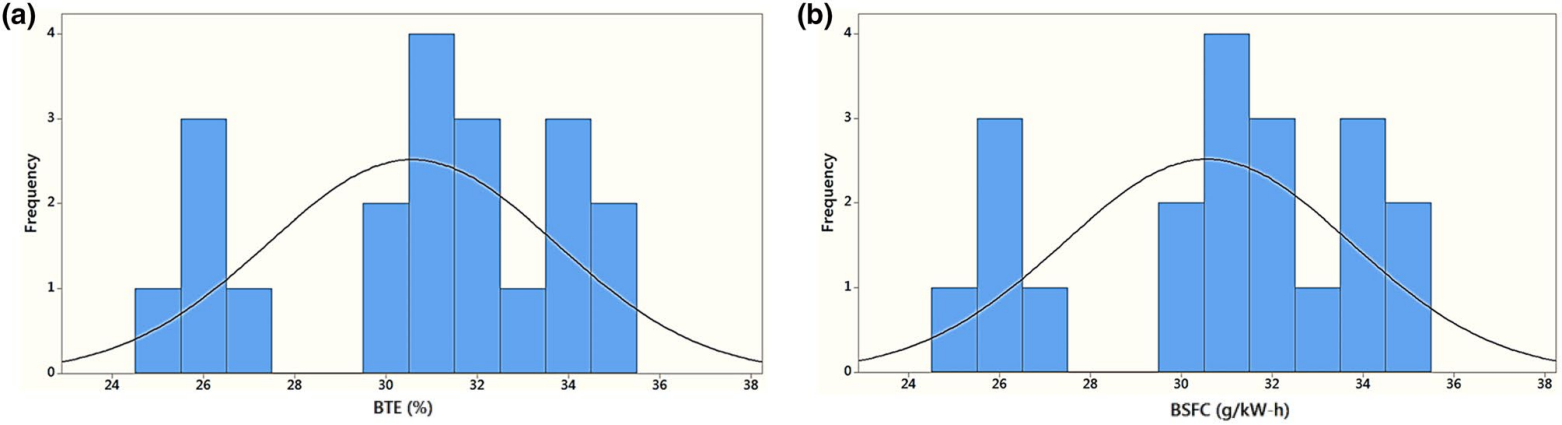
Variation of frequency with: (**a**) brake thermal efficiency and (**b**) brake specific fuel consumption.

Table 3 shows the analysis of variance (ANOVA) of the engine parameters of IOP and biodiesel blend that effect BTE. The error involved was only 1. Hence, the parameters required majorly contributed to the engine performance. The degree of freedom (DF) was 4 for injection pressure and 6 for the blend. The adjusted sum of squares (SS) showed that the blend contributed significantly to the main effect of BTE i.e., 2,265.76. The combined effect of IOP and blend was minimal. The mean square (MS), F-value, and *P* value indicated the same level of effect of pressure and blend on BTE, as indicated by adj. SS. The main effect on the mean of BSFC is shown in Fig. 5. IOP indicated that the main effect on the increase in values and it reduces fuel consumption. Later, for IOP of 1,000 bar, fuel consumption increases as explained previously concerning Fig. 6. The fuel consumption for diesel is low and exponentially increases with the addition of blend. The blends 1–4 indicate diesel, D10030, MOME20, and MOME2030, respectively.

**Table 3:** Analysis of variance showing the level of parameters effect on BTE.

Source	*df*	Adj SS	Adj MS	F-value	*P* Value
Model	5	3,759.27	751.85	1.72	0.195
Linear	2	2,835.78	1,417.89	3.25	0.070
Injection pressure	4	570.02	570.02	1.30	0.273
Blend	6	2,265.76	2,265.76	5.19	0.039
Square	2	887.36	443.68	1.02	0.387
IOP * IOP	1	407.16	407.16	0.93	0.351
Blend * Blend	1	480.20	480.20	1.10	0.312
2-way interaction	1	36.12	36.12	0.08	0.778
IOP (bar) * Blend	1	36.12	36.12	0.08	0.778

Figure 6 shows the histogram of BSFC and BTE frequency at different ranges. Figure 4a depicts that the BTE percentage obtained in this study is the average range of 30–32%. Similarly, the BSFC indicating the amount of fuel consumed is the highest in the average range. BTE of 31% appeared four times in the 20 readings observed. The peak at the center of the curve indicated a high occurrence of BTE and BSFC in the mid-range. In Table 4, the statistics of BTE and BSFC has been presented considering the effect of IOP and blends. The mean, standard deviation, and Q1/Q2 values are mentioned have indicated that the deviation from the mean was high, showing, more substantial influence of parameters involved. The Q1 value indicated the mid-value of the first half, and Q2 reported the mid of the second half.

**Table 4:** Descriptive statistics of BTE and BSFC obtained for different injection pressure and blend.

Variable	N	N*	Mean	SE mean	SD	Minimum	Q1	Median	Q3	Maximum
BTE (%)	20	0	30.572	0.709	3.173	25.000	27.512	31.000	33.275	35.000
BSFC (g/kW-h)	20	0	201.70	5.10	22.80	160.00	182.50	207.50	217.25	240.00

### Effect of IOP on engine emission characteristics

**Figure 7:**
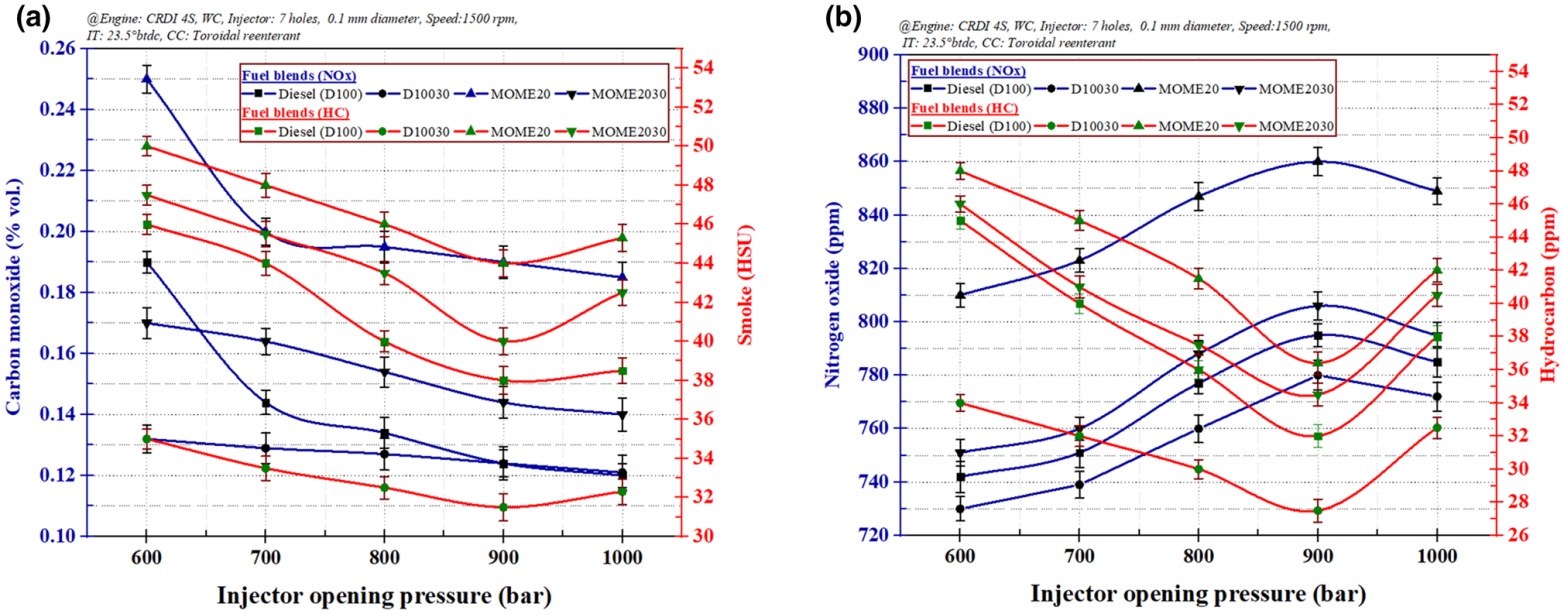
The variation of injection opening pressure: (**a**) Carbon monoxide and smoke emissions, (**b**) Nitrogen oxide and hydrocarbon emissions.

The variations of carbon monoxide (CO) and smoke emissions at 80% load and 7-hole FI at different IOP are shown in Fig. 7a. Increasing the injector opening pressure ensured uniform mixing of the air–fuel ratio near the stoichiometric condition. This occurrence was suitably checked with the measurements of both air and fuel flow rates to ascertain the air–fuel ratio that was a chemically correct mixture for different loading conditions^50^. At 80% engine loading operation with selected fuel combinations, the air–fuel ratio was varied from 16.84 to 22.97%, which suggests appropriate stoichiometric mixture condition. Also, the nozzle hole number reduced the CO emission under stoichiometric conditions^51^. The diesel fuel emitted lower CO with the addition of 30 ppm ZnO nanoparticles because the increase in injection pressure raised the temperature of combustion and pressures due to proper A:F mixing and thus completely utilizing available air leading to improved combustion^25, 52^. The lower BTE of biodiesel was the primary reason behind increased emissions from the CRDI engine even at higher pressures^53^. For the fuel blend MOME20, the addition of ZnO nanoparticles at all injection pressure has illustrated reductions in CO and HC emission. Figure 7b represents the NOx and HC emissions of the CRDI engine at varying IOP for different fuel blends. The higher NOx emission from the MOME20 blend compared to diesel at all pressures is due to the intense combustion reaction^53^. The diesel emits lower NOx thus addition of ZnO nanoparticles further slightly reduced the NOx. Also, the ZnO nanoparticles lower the pre-mixed burn fractions in the combustion chamber due to lower ignition delay period and thus, facilitates in reducing the combustion temperature^19, 54^.

A similar trend was observed for HC and CO emission with increasing IOP. At a higher IOP of 900 bar, small fuel droplet size resulted in reduced smoke emissions. Furthermore, the addition of 30 ppm of ZnO nanoparticles in diesel and MOME20 reduces the smoke emissions. Also, the reduction is attributed to the effect of 2% DEE, which improves the cetane number and results in complete fuel combustion, thereby reducing the emissions^55^.

#### The effect of injection timing (IT) on engine characteristics

##### The effect of injection timing (IT) on engine performance characteristics

**Figure 8:**
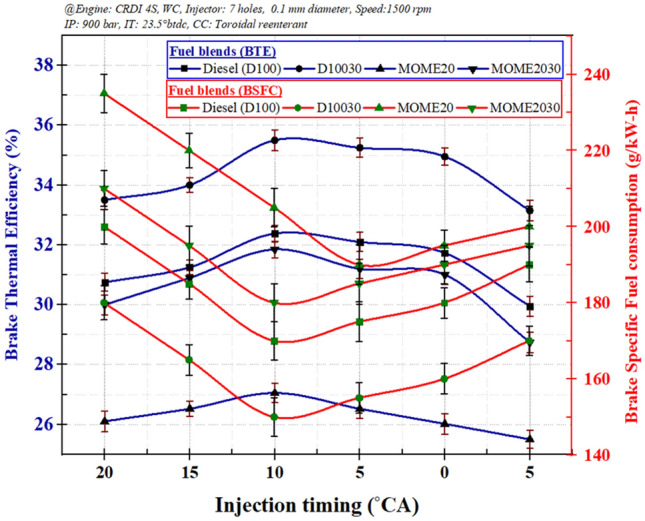
The variation of injection timing with brake specific fuel consumption and brake thermal efficiency.

Figure 8 illustrates the variation of BSFC and BTE for injection timing (IT) from 20°CA to 5°CA for 7-hole fuel injector at 80% load. The BTE initially reduced due to the higher fuel consumption and gradually increased owing to delayed injection angle resulting in lower fuel consumption^27^.

The IT of 10°CA illustrated the maximum reduction and enhancement in BSFC and BTE, respectively, for all fuel blends. The fuel blends, D10030 and MOME2030, demonstrated 11.7% and 12.2% reduction, respectively in fuel consumption as compared to diesel and MOME20. Also, the BTE increases with the addition of ZnO nanoparticles by 9.6% and 16.4% for D10030 and MOME2030, respectively, in contrast to diesel and MOME20 due to the enhanced micro explosion phenomenon^56^. Besides, the reduction in fuel consumption is attributed to the enhanced squish in TRCC that facilitates improvement in the swirl rate; similar observations were reported in preceding literature^27, 57^.

##### The effect of injection timing (IT) on engine emission characteristics

**Figure 9:**
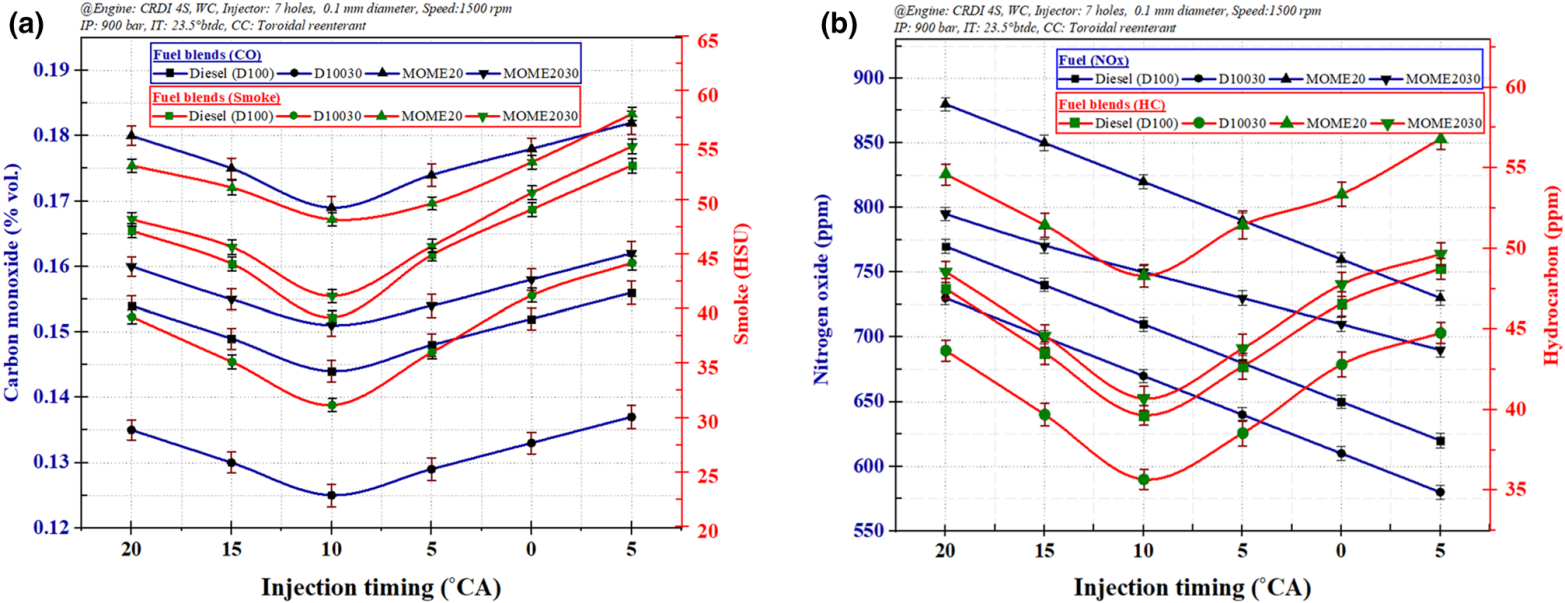
The variation of Injection timing: (**a**) Carbon monoxide and Smoke emissions and (**b**) Nitrogen oxide and Hydrocarbon emissions.

Figure 9a,b illustrate variations of CO and smoke, and NOx and HC emissions at varying IT for 7-hole FI and TRCC. The zinc oxide nanoparticles in diesel fuel (D10030) provides extra oxygen molecules, enhances the micro-explosion phenomenon, and increases overall combustion characteristics^43, 46, 58^. The CO emissions were slightly higher at 20°CA and 15°CA due to incomplete combustion of fuel blends, the rise in the delay results in the accumulation of unburned HC in the engine cylinder^27^. At 10°CA, due to the better utilization of air, lower penetration distance reduced wall impingement, and mass flow rate, which lowers the emissions^27, 59^. If the pilot fuel injection occurs too early, it forms a lean mixture increasing fuel consumption rate^27^. An enhanced air movement in TRCC and supply of higher oxygen molecules through the addition of ZnO nanoparticles and MOME20 results in improved fuel combustion compared to MOME20 resulting in a reduction in CO and HC emissions by 10.6% and 15.7% for MOME2030 fuel blend. The MOME20 blend influences the combustion and emission process and leads to the slow development of spray that leads to poor atomization and evaporation owing to the improper injection. The factors influencing the NOx are flame temperature, injection timing and fuel properties^60^. The premixed combustion phase results in the NOx formation from burned gases developed from combustion adjacent to stoichiometry and lean flammable mixtures^57^.

The fuel from the pilot-injection initiates combustion, the temperature and pressure being higher in the cylinder lead to rapid fuel combustion of injected fuel during the main injection. This injection restrains sharp intensification of pressure during the rapid combustion phase and eventually reduces knocking and subsequently producing NOx. An added rationale for the rise in the NOx may be attributed to the fact that a more substantial part of the combustion is achieved before TDC for MOME20 and its blends as compared to diesel and nano-diesel blends due to lower ignition delay^41, 61^. The maximum thermal efficiency of nano-additives enhances the combustion phenomenon through an increase in the convective heat transfer coefficient^1, 43^. Also, 2% DEE improved combustion efficiency. Thus less fuel was burned, resulting in lower emissions^55, 62^.

### Filter blocking tendency (FBT)

The premature clogging of diesel fuel filter has increased significantly during the last decade due to the overuse of biodiesel in diesel engines, cold weather conditions, contaminant formation, solvency characteristics of the base diesel, and use of high-pressure common-rail (HPCR) engines. This clogging results in a longer delay period, poor drivability, and increased maintenance in various fuel filter applications. Also, the restrictions in the fuel filters pore size, small clearances in HPCR injectors, uneven nanoparticle size, and carboxylate salts in the fuel are known to accelerate the diesel fuel filter plugging^63^. The FBT helps characterize the influence of various fuels and additives on the fuel filtration unit. The FBT was analyzed in accordance with the ASTM D2068-17 standards.

In the present study, FBT estimation was adopted from the previous research by Alexandra S. Fersner et al.^64^. Initially, 300 ml of the fuel was pumped through a 1.6 μm pore size glass fiber filter (Whatman, GF/A) at a rate of 20 ml/min. After 300 ml of the fuel passes through the glass fiber filter, the final pressure was observed, and the FBT was calculated using Eq. 4. The test was complete when the entire 300 ml of fuel was pumped through the glass fiber filter, and the pressure reaches a value of 105 kPa

4 $${\text{Filter}}\,{\text{blocking}}\,{\text{tendency}} = \sqrt {1 + \left( \frac{P}{100} \right)^{2} }$$

where ‘P’ is the maximum pressure obtained in kPa (values range between 1 and 1.41).

**Figure 10:**
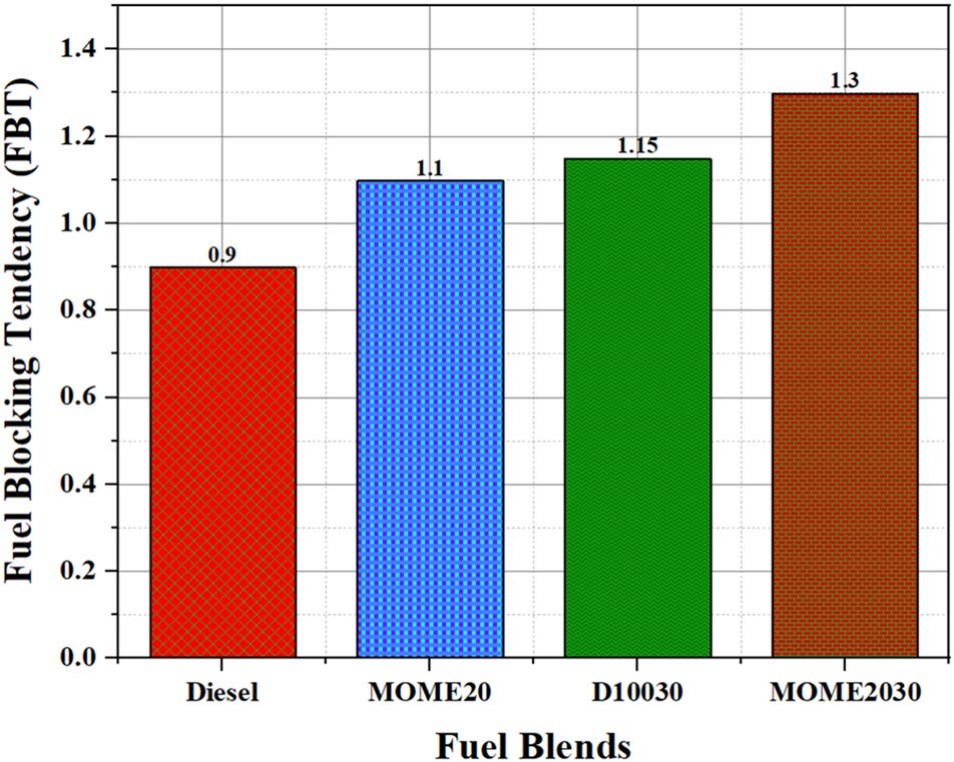
Filter blocking tendency (FBT) of fuel blends.

The FBT values of the fuel blends measured using Multi Filtration Tester (MFT, model: 10-325-000) illustrated decent filtration properties. The biodiesel and the nano additives slightly increased the FBT values due to high viscosity. However, the nano size of the zinc oxide additives ensured the passage of nanoparticles through micrometer glass fiber filter. The FBT results of test fuels are shown in Fig. 10; the results illustrate values for all the nano fuel blends; it lies within the permissible limit of 1.4. Therefore, the nano zinc oxide can be used as a fuel additive in diesel and biodiesel fuel blends functions without any risk of fuel filter clogging.

## Conclusions and future prospects

The application of diesel, MOME20, D10030, and MOME2030 fuel blends on the CRDI engine illustrated that the modification of injector nozzle holes significantly influenced the process of combustion, combustion chamber piston bowl geometry and nano additives in fuel blends. Based on the obtained results the conclusions are drawn.

1. At 900 bar IOP and 10°CA IT, the BTE for D10030 and MOME2030 for 7-hole injectors and 80% load increased by 7.3% and 18.7%, and 9.7% and 16.4%, respectively. Also, the BSFC reduced by 17.1% and 3.2%, and 11.8% and 12.2% at 900 bar IOP and 10°CA IT for D10030 and MOME2030 when compared to diesel and MOME20. The increase in the BTE is due to the enhanced squish, swirl, and turbulence of air and fuel particles and catalytic effect of ZnO nanoparticles and DEE additives.
2. The combined effect of 7-hole fuel injector, TRCC and 30 ppm of zinc oxide nanoparticles in diesel and biodiesel fuel blends reduced the smoke, CO, HC and NOx emissions by 20.6%, 13.2%, 10.1% and 5.7% for D10030 and 14.59%, 10.65%, 15.73% and 8.53% for MOME2030 at 10°CA IT and 17.2%, 2.1%, 14.1%, 1.9% for D10030, and 9.1%, 24.2%, 5.2% and 6.3% for MOME2030 at 900 bar IOP respectively.
3. The nanofuel blends results in higher combustion chamber wall temperature provides more oxygen and enhances the air and fuel mixture due to the improved air swirl. Also, the 7-hole fuel injector and modified injection timing delivered enough time for preparation and mixing of fuel charge before the start of injection resulting in lower emissions and fuel consumption. Therefore, the cylinder pressure and heat release rate for D10030 and MOME2030 improved by 6.8% and 17.1%, and 7.4% and 12.3% respectively compared with the other two counterparts.
4. The fuel blocking tendency (FBT) analysis illustrated the nano fuel blends to be within the permissible limit of 1.4. The zinc oxide nanoparticles of 22.5 nm size do not obstruct the fuel filter, and these nanofuel blends led to smoother engine operation.

The results and conclusions validated that the toroidal reentrant combustion chamber, 7-hole fuel injector, and zinc oxide nano-additive blended with DEE, CTAB surfactant and diesel-Mahua biodiesel at 900 bar IOP and 10°CA IT enhanced the engine combustion, improved the performance characteristics and lowers the emission in CRDI diesel engine.

According to current and preceding studies, there is a scope for further studies on the modification of fuels and engines:

1. The investigation of surface reaction on engine parts such as cylinder linings, exhaust pipe, combustion chamber, fuel injectors, piston, and piston rings are necessary to validate the reliability of nano-additives in a diesel engine^3, 11^.
2. A more in-depth sustainability analysis can be presented using enhanced sustainability assessment tools such as exergy, exergo-economic, and exergo-environmental analyses^65, 66^. Also, study on hybrid nano catalyst in fuel blends and exergetic performance of diesel engines can be developed^67, 68^.
3. The complexity in the synthesis of nanoparticles and economic viability should be considered in future research. Also, a study on developing cost-effective nano additives is essential^2, 3^.
4. The effect of metal-based nanoparticles used as fuel additives in diesel/biodiesel fuel on human health and environmental pollution is required before the commercialization of the technology^69^.
5. Development of different fuel injectors and piston bowl geometries to further enhance and boost the turbulence, swirl, squish, air to fuel ratio, and overall engine characteristics.

## Experimental

In this section, preparation of, nanofuel blend preparation, experimental setup, properties of nanofuel blend, and uncertainty analysis are explained in detail.

### Preparation of Mahua biodiesel

The Mahua biodiesel was prepared using a transesterification reaction; the method was adopted from the previous investigations by Soudagar et al.^1, 2, 70^. The Mahua oil (*Madhuca indica*) with high free fatty acid content above 20% and an acid value of 39.8 mg KOH/g was used in the present study. The acid value (AV) was well above the acceptable limits for the transesterification reaction using an alkaline catalyst. Therefore, to reduce the acid value of *Madhuca indica* lower than 2 mg KOH/g, sulfuric acid (H_2_SO_4_: 1% v/v) was used for the conversion of FFAs to esters. Later, the Mahua oil was heated on a magnetic heater and stirrer at a temperature of 60–65 °C then 1:6 ratio of methanol (CH_3_OH) was added. Finally, 3 gm/l of oil of Sodium hydroxide was added as a catalyst to the biodiesel mixture. The experiments were carried out using a setup consisting of a 500-cc glass flask sealed using airtight caps to prevent methanol evaporation. For proper blending, the mixture was stirred continuously and kept constant for 48 h for the reaction mixture to settle. A distinct line was observed indicating Mahua biodiesel and glycerol as a by-product of the reaction. The oil being lighter than glycerol it floats at the top. The methyl ester of Mahua was extracted and rinsed with warm water to get rid of any existing contaminations such as suspended particles, excess methanol, or sulfuric acid molecules. The washed mixture is kept for settling for another 40 h. The oil was gently transferred to a beaker and heated at 60–65 °C to remove any trace of excess water.

### Preparation of nano fuel blends

**Figure 11:**
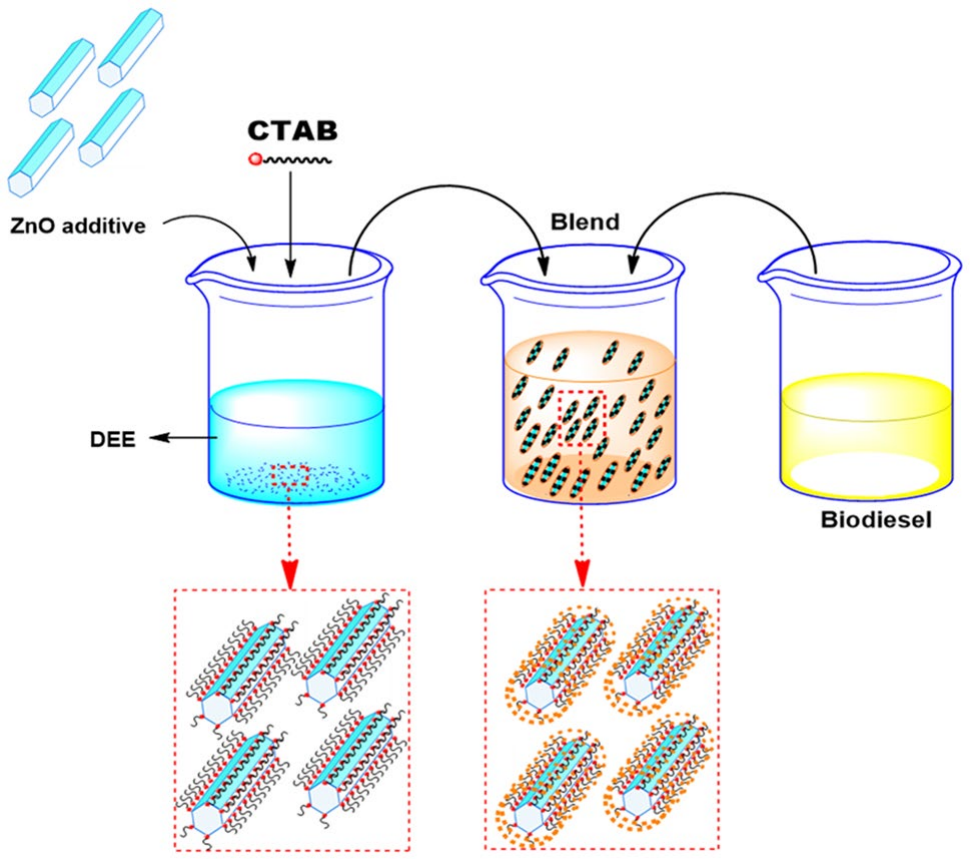
Schematic diagram of the preparation of nanofuel blend.

The preparation of nanofluids should satisfy three stability aspects viz. kinetic stability, dispersion stability, and chemical stability for the nanofluids to be free from agglomeration for a longer duration^3, 11, 71^. The two-step ultrasonication process was used in the dispersion of nanoparticles into the base fluid. The zinc oxide NPs were dispersed in distilled water using a bath-type sonicator, which is an indirect method of sonication, the ultrasonicator operates at a frequency higher than 20 kHz for 30 min. The sonication was used to enhance the dissolution, by breaking intermolecular interactions to avoid clustering, agglomeration, and settling^3, 72, 73^. Also, the ultrasonication process facilitated the agglomerated nanoparticles back to their nanometer range and used to remove dissolved gases from liquids by sonicating the fluid under vacuum. The D10030 sample consists of 100% diesel, 30 ppm ZnO nanoparticles, and 10 mg CTAB surfactant, and MOME2030 contains 80% diesel, 20% Mahua biodiesel, 30 ppm ZnO nanoparticles and 10 mg CTAB, and 2 vol.% of DEE. Figure 11 illustrates the schematic representation of the preparation of the nanofuel blend. Furthermore, the probe sonicator is used for a steady blending of nanoparticles in the fuel; the ultrasound waves are supplied every 10–30 s at 25 kHz for 20 min.

### Test engine setup

**Figure 12:**
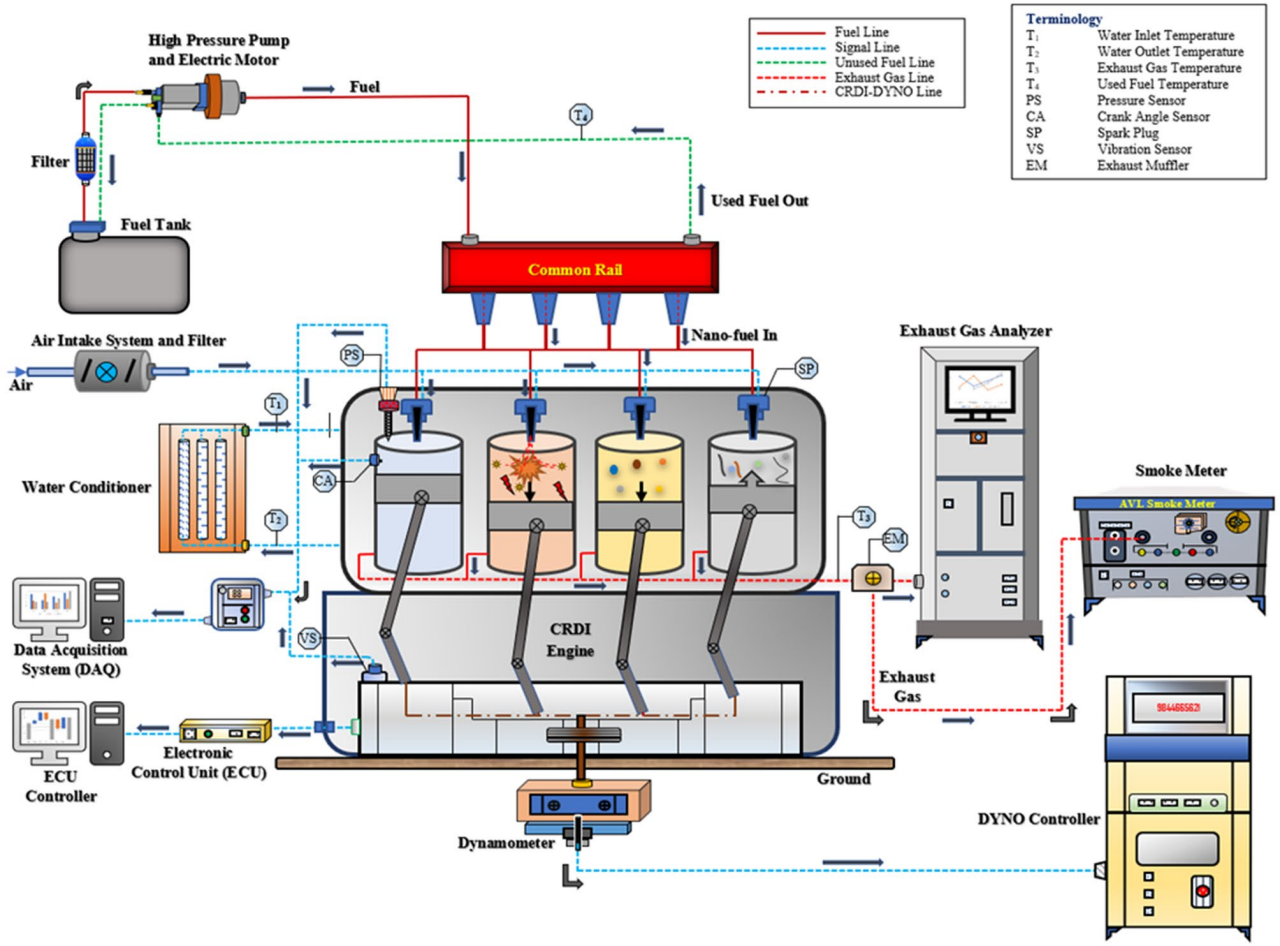
Schematic layout of computerized common rail direct injection (CRDI) diesel test engine.

The engine trials were observed through Dynomax-2000, a software data control system. AVL DiSmoke 4000 gas analyzer was used to monitor the readings of smoke opacity. Eddy current dynamometer was used to employ the loading. Figure 12 demonstrates the four stokes of a CRDI diesel engine.

Then, several lengthy CRDI engine tests were conducted using diesel, D10030 (100% Diesel + 30 ppm ZnO nanoparticles), MOME (80% Diesel + 20% MOME) and MOME2030 (80% Diesel + 20% MOME + 30 ppm ZnO nanoparticles). The internal air jets were converted to toroidal reentrant type leading to the revolution of the air and fuel charge about the cylinder axis referred to as swirl. The swirling motion arises during suction stroke and significantly improves throughout the compression stroke in bowl-in-piston combustion chambers (TRCC) designs. The TRCC results in a significant increase in swirling motion at the end of the compression stroke^60^. The test results for a reentrant combustion chamber shape demonstrated a considerable enhancement in diesel engine characteristics due to the protruding lip, which is almost equal to the bowl height compared to the open straight sided bowl designs^74^. The present study deals with the modification of fuel, combustion chamber, and fuel injector to attain the maximum potential of biodiesel in the CRDI engine.

## List of symbols

ppm: Parts per million
nm: Nanometre
°CA: Crank angle (°)
°C: Celsius
g: Gram
g/kwh: Gram per kilowatt hour
h: Hour
kg: Kilograms
kJ/kg: Kilo Joules per kilogram
kW: Kilowatt
mg: Milligram
min: Minutes
m: Meter
mm: Millimeter
MPa: Mega Pascal
N-m: Newton meter

## Abbreviations

NO_X_: Oxides of nitrogen
CO_2_: Carbon dioxide
HC: Hydrocarbon
CO: Carbon monoxide
A:F: Air–fuel mixture
MOME20: 20% Mahua oil biodiesel blended with diesel
D10030: Diesel with 30 ppm ZnO NPs
TEM: Transmission electron microscopy
SAED: Selected area (electron) diffraction
HRTEM: High-resolution transmission electron microscopy
CI: Compression ignition
NPs: Nanoparticles
ZnO: Zinc oxide
DEE: Diethyl ether
HRR: Heat release rate
ID: Ignition delay
BSFC: Brake specific fuel consumption
BTE: Brake thermal efficiency
CC: Combustion chamber
HCC: Hemispherical combustion chamber
TRCC: Toroidal re-entrant combustion chamber
TCC: Toroidal combustion chamber
BTDC: Before top dead centre
ATDC: After top dead centre
CR: Compression ratio
SOI: Start of injection
PP: Peak pressure
CD: Combustion duration
IP: Injection pressure
IT: Injection timing
CTAB: Cetyl trimethyl ammonium bromide
FI: Fuel injector
MOME: Mahua oil methyl ester (Mahua biodiesel)
MOME2030: MOME20 with 30 ppm ZnO NPs
SEM: Scanning electron microscopy
FTIR: Fourier transform infrared spectroscopy
XRD: X-ray diffraction
EDX: Energy dispersive X-ray
